# Innate Immune Response to SARS-CoV-2 Infection: From Cells to Soluble Mediators

**DOI:** 10.3390/ijms22137017

**Published:** 2021-06-29

**Authors:** Daniela Ricci, Marilena Paola Etna, Fabiana Rizzo, Silvia Sandini, Martina Severa, Eliana Marina Coccia

**Affiliations:** Department of Infectious Diseases, Istituto Superiore di Sanità, 00161 Rome, Italy; daniela.ricci@iss.it (D.R.); marilenapaola.etna@iss.it (M.P.E.); fabiana.rizzo@iss.it (F.R.); silvia.sandini@iss.it (S.S.); martina.severa@iss.it (M.S.)

**Keywords:** SARS-CoV-2, innate immunity, soluble and cellular mediators

## Abstract

The vulnerability of humankind to SARS-CoV-2 in the absence of a pre-existing immunity, the unpredictability of the infection outcome, and the high transmissibility, broad tissue tropism, and ability to exploit and subvert the immune response pose a major challenge and are likely perpetuating the COVID-19 pandemic. Nevertheless, this peculiar infectious scenario provides researchers with a unique opportunity for studying, with the latest immunological techniques and understandings, the immune response in SARS-CoV-2 naïve versus recovered subjects as well as in SARS-CoV-2 vaccinees. Interestingly, the current understanding of COVID-19 indicates that the combined action of innate immune cells, cytokines, and chemokines fine-tunes the outcome of SARS-CoV-2 infection and the related immunopathogenesis. Indeed, the emerging picture clearly shows that the excessive inflammatory response against this virus is among the main causes of disease severity in COVID-19 patients. In this review, the innate immune response to SARS-CoV-2 infection is described not only in light of its capacity to influence the adaptive immune response towards a protective phenotype but also with the intent to point out the multiple strategies exploited by SARS-CoV-2 to antagonize host antiviral response and, finally, to outline inborn errors predisposing individuals to COVID-19 disease severity.

## 1. Introduction

The severe acute respiratory syndrome coronavirus 2 (SARS-CoV-2) is the etiologic agent of the coronavirus disease 2019 (COVID-19) pandemic first reported in Wuhan, China, in December 2019. To date, as of 20 June 2021, a total of 179,127,481 cases and a total of 3,879,122 deaths have been confirmed (worldometers.info/coronavirus).

SARS-CoV-2 belongs to the Betacoronavirus genus, which also includes OC43 and HKU1 (which can cause the common cold in an immunocompetent host) of lineage A, SARS-CoV of lineage B, and MERS-CoV of lineage C [1,2]. SARS-CoV-2 genome analysis revealed a 79.5% and 51.8% sequence identity with SARS-CoV and MERS-CoV, respectively. Given the great genomic similarity with SARS-CoV, SARS-CoV-2 is classified as a member of lineage B [2]. SARS-CoV-2 also displays a 96.2% identity with the bat CoV RaTG13, thus strongly pointing to the bat as the original and natural host of the virus prior to human transmission through spillover via an undetermined intermediate host [3].

SARS-CoV-2 is a positive-sense single-stranded (ss)RNA virus, whose genome comprises an open reading frame (ORF)1a/b at 5′-terminus that encodes two polyproteins, pp1a and pp1ab. After proteolytic cleavage, these polyproteins result in 16 nonstructural proteins (nsp). The 3′-terminus of the SARS-CoV-2 genome encodes four viral structural proteins, including the spike (S) glycoprotein, the envelope (E) protein, the membrane (M) protein, and the nucleocapsid (N) protein. Furthermore, the 3′-terminus contains nine accessory proteins, namely Orf3a, Orf3b, Orf6, Orf7a, Orf7b, Orf8, Orf9b, Orf9c, and Orf10 [4]. The virion surface is composed by both M and E proteins, crucial for intracellular membrane assembly and efficient production and release of virions, as well as the S protein, responsible for viral entry into the host cells through direct binding to the host cell receptor and membrane fusion. Interestingly, subversive activities on host immune response have been reported for both SARS-CoV-2 structural, nsp, and accessory proteins [2].

Throughout the last year, a significant number of key questions have been addressed regarding immune response to SARS-CoV-2 infection, whose answers have been and will be helpful for the comprehension of several not yet solved issues on the viral immunopathogenesis and, in turn, for the development of therapeutic and prophylactic strategies. Nonetheless, how innate immunity tunes the fate of SARS-CoV-2 infection and how long a protective memory response against the virus will last represent critical aspects to be investigated. Considering the broad and rapidly evolving knowledge on innate and adaptive immunity against SARS-CoV-2, the main focus of this review is to provide a comprehensive view on the current understanding of the early immune response to SARS-CoV-2, including both soluble mediators and cellular components involved in the interaction of this virus with the host immune system. Furthermore, a description of the viral immune evasion strategies and the innate inborn immunodeficiencies contributing to COVID-19 severity will be provided. We apologize to those colleagues whose data and contributions to this field have been overlooked due to space or our knowledge limitations.

## 2. Host–Pathogen Interaction

Host immune response and cell tropism are the major determinants influencing the fate of SARS-CoV-2 infection [5,6]. Similarly to SARS-CoV, SARS-CoV-2 entry into host cells is mediated by the interaction between viral S glycoprotein with the angiotensin-converting enzyme 2 (ACE2) receptor expressed on the surface of different cell types, including the surfactant producing type 2 alveolar cells and the ciliated and goblet cells in the airways [5,7]. Each monomer of the trimeric S glycoprotein contains two subunits, S1 and S2. The S1 subunit is responsible for the cellular tropism of the virus and includes the key functional receptor binding motif within the receptor binding domain, while the S2 subunit mediates the fusion between virus and cell membranes [5]. Following receptor binding, the priming of the S protein is mediated by the transmembrane serine protease 2 (TMPRSS2), a host cell transmembrane serine protease, that entails the S protein cleavage at the S1/S2 and S2 sites. The enzymatic processing favors the fusion among viral and cellular membranes, thus allowing the release of viral ssRNA into the cytoplasm [8,9,10]. Although to date ACE2/TMPRSS2 function in SARS-CoV-2 entry has been widely demonstrated, few studies have suggested that other cellular proteases and cofactors might have a role, especially in the case of the infection of cells expressing low ACE2/TMPRSS2 levels [11,12]. Indeed, it has been reported that in cells TMPRSS2^−^ or with low TMPRSS2 expression, the cysteine protease cathepsins B and L and furin might synergize in promoting viral entry [13]. Moreover, neuropilin-1 has been described as a facilitator of SARS-CoV-2 cell entry and infectivity [11]. A comprehensive picture of the SARS-CoV-2 receptor repertoire and related molecules is given by Radzikowska et al., who performed RNA sequencing of different primary cells and tissues [14]. Interestingly, this analysis showed that, differently from epithelial cells, immune cells do not express ACE2 or TMPRSS2, while they do express CD147, another receptor for SARS-CoV-2, thus providing an additional route for viral entry [14].

Once released into the cytoplasm, viral RNA is translated into polyproteins composing the replicase-transcriptase complex [15]. This complex transcribes a negative-sense RNA intermediate that works as a template for the synthesis of newly genomic and subgenomic positive-sense RNAs. In doing so, double-stranded RNAs (dsRNA) are produced early on during the infection cycle as a result of genome replication and mRNA transcription. Then, the synthesis of proteins contributing to viral function and assembly, including M, S, and E, occurs in the endoplasmic reticulum-Golgi compartment (ERGIC). Finally, the nucleocapsid, generated by the association of N protein with the newly synthetized genomic RNA, reaches the ERGIC where it combines with SARS-CoV-2 structural proteins to produce the viral progeny for later export in the extracellular space through exocytosis [16].

As mentioned above, the canonical SARS-CoV-2 entry receptor, ACE2, is mainly expressed on ciliated and goblet cells in the airways and on a cell subset in the lung named surfactant producing type 2 alveolar cells (type II pneumocytes). Accordingly, ciliated and goblet cells constitute a portal for the entry of SARS-CoV-2, which then reaches the upper respiratory tract. However, since a proportion of patients showed extrapulmonary symptoms, it is likely that SARS-CoV-2 can infect a wide range of cells expressing ACE2 receptor from other organs such as heart, kidney, testis, eye, endothelium, and intestinal epithelium [17].

A variety of clinical manifestations characterizes SARS-CoV-2 infection ranging from asymptomatic to mild, moderate, and severe disease. The great majority of infected individuals (80%) are asymptomatic or show mild symptoms, while about 15% progress to severe symptomatology. The remaining 5% of patients develop acute respiratory distress syndrome (ARDS), septic shock, and multiorgan failures accompanied by a high risk of death, in particular for the elderly population [18,19].

Reasoning on the variable human-to-human response to SARS-CoV-2 infection, it is evident that disease severity not only depends on viral infection but is deeply influenced by host immune response. Indeed, in severe COVID-19 disease, diffuse airway injury and impaired alveolar gas exchange are sustained by high levels of circulating cytokine and chemokine, the so-called “cytokine storm”, exacerbated inflammatory infiltrate in pulmonary tissue, and profound lymphopenia.

## 3. Innate Immune Response to SARS-CoV-2

An efficient immune response against invading pathogens requires the early activation of innate immunity, a nonspecific response able to control infection by means of antiviral and proinflammatory molecules and to sustain the specific adaptive immunity that contributes to clearing the infection and preventing reinfection by the same pathogen. In the case of SARS-CoV-2, the recognition by tissue-resident immune cells within the lung provides a local immune response resulting in the recruitment of further innate immune cells from the blood.

**Figure 1:**
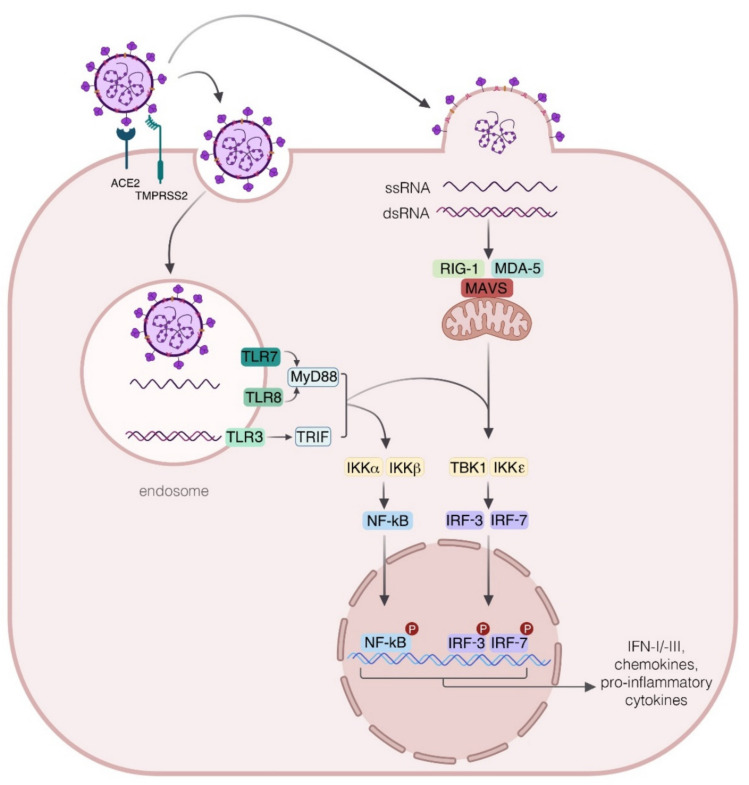
Innate immune response to SARS-CoV-2. The interaction of SARS-CoV-2 with targeted cells requires the recognition of viral spike (S) protein by the angiotensin-converting enzyme (ACE2) and the priming of the S protein by the host cell transmembrane serine protease 2 (TMPRSS2). SARS-CoV-2 can gain access to host cells by means of the endocytic pathway that allows virus localization into the endosomes, where the viral single-stranded (ss) RNA is detected by the endosomal Toll-like receptors (TLR)7 and 8. Soon after viral RNA recognition, the adapter protein myeloid differentiation factor 88 (MyD88), is engaged for the activation and nuclear translocation of transcriptional factors nuclear factor kappa light-chain-enhancer of activated B cells (NF-κB) and interferon regulatory factor-3 (IRF-3) and 7 (IRF-7) for the transcription of genes encoding for proinflammatory cytokines and type I and III interferons (IFNs). In addition, TLR3 can be activated by double stranded (ds) RNA, produced during viral replication, thus contributing via the TRIF pathway to a protective response in SARS-CoV-2 infection. The sensing of replicating virus also occurs by the cytosolic receptor retinoic acid-inducible gene I (RIG-I) and the melanoma differentiation-associated protein (MDA5), which in turn recruits the mitochondrial antiviral signaling protein (MAVS), then leading to the IRF-3 and IRF-7 phosphorylation required for the expression of IFNs. IKKα: I-Kappa-B Kinase Alpha; IKKβ: I-Kappa-B Kinase Beta; IKKε: I-Kappa-B kinase epsilon; TBK1: TANK binding kinase 1; TRIF: TIR domain-containing adapter protein inducing interferon (IFN)-β.

To establish an antiviral response, host cells must recognize specific pathogens associated with molecular patterns such as nucleic acids and proteins via specific pattern recognition receptors (PRRs). RNA genomes, including those of SARS-CoV, MERS-CoV, and SARS-CoV-2, can be detected by endosomal/lysosomal PRRs, such as Toll-like receptors (TLR) 3, 7, and 8, or by cytosolic RNA sensors, namely the retinoic acid-like receptors that encompass retinoic acid-inducible gene I (RIG-I) and melanoma differentiation-associated protein 5 (MDA5), and, in turn, trigger antiviral response through the mitochondrial antiviral signaling protein (MAVS) activation [20,21] (Figure 1). The viral RNA sensing by TLR promotes the recruitment of Toll-interleukin receptor (TIR) domain-containing adapter proteins, such as myeloid differentiation factor 88 (MyD88), TIR domain-containing adapter protein (TIRAP), and TIR domain-containing adapter protein inducing interferon (IFN)-β-related adapter molecule (TRIF), leading to the activation of the transcription factors IFN regulatory factor-3 (IRF-3), IRF-7, and nuclear factor kappa light-chain-enhancer of activated B cells (NF-kB) required for the transcriptional induction of the antiviral IFN, proinflammatory cytokines, and chemokines [22,23,24,25]. This scenario generally occurs in SARS-CoV-2-infected individuals with asymptomatic or mild upper respiratory illness, who efficiently control infection. Nevertheless, immune responses to SARS-CoV-2 sometimes unpredictably deviate towards inflammatory tissue damage, leading to rapid evolution from moderate to severe disease characterized by progressive pneumonitis, ARDS, and multiorgan failure with fatal outcomes. Both soluble and cellular mediators contribute to these two opposite outcomes, although the mechanisms that tilt the balance between the protective and deleterious immune response are not yet fully understood. Moving from the evidence that innate immune response is crucial in determining the fate of COVID-19 pathogenesis, here we focus our attention on the main actors involved from the early phases of SARS-CoV-2 infection and whose activity is closely interlinked, namely the IFNs, proinflammatory cytokines, and innate immune cells. In the most favorable scenario, a quick IFN production occurring soon after infection from the infected cells limits viral replication within a few days with the help of innate immune cellular actors [26]. Conversely, in the absence of a robust and rapid antiviral response that might be due to virus-triggered immune evasion strategies or to pre-existing medical and/or genetic conditions, the ongoing infection can promote an exaggerated production of cytokines and chemokines, leading to the activation and the recruitment of different immune cell subsets with subsequent local tissue damage. When the cytokine storm becomes unstoppable, chronic or irreversible end-organ dysfunctions may occur, also leading to death.

## 4. Soluble Mediators

### 4.1. Interferon Production

The protective innate immune response against the virus at the early stage of infection requires a robust IFN production. The IFN family is divided into three main groups: type I (mainly IFN-α/β), type II (IFN-γ), and type III (IFN-λ) IFNs [25]. In particular, type I IFNs are secreted by all nucleated cells after virus infection, in addition to the specialized IFN-producing plasmacytoid dendritic cells (pDC) that can also sense the presence of viruses in the absence of productive viral replication [26]. Type II IFN or IFN-γ is principally produced by NK cells and T helper 1 (Th1) cells and plays a pivotal role in tuning the innate and adaptive immunity against several microbes. IFN-λs share similar functions and intracellular pathways with type I IFNs, although differences exist with respect to their regulation and signaling outputs [27].

Once released by the infected cells, type I IFNs bind to the dimeric IFN receptors (IFNAR) composed by IFNAR1 and IFNAR2 subunits and, through the activation of tyrosine kinase 2 and Janus kinase 1, mediate the phosphorylation of the signal transducers and activators of transcription (STAT)1 and STAT2. Phosphorylated STATs heterodimerize with IRF-9 to form the IFN-stimulated gene factor 3 complex that, after nuclear translocation, initiates the transcription of IFN-stimulated genes (ISGs) through its binding to IFN-stimulated response elements (ISREs) within their gene promoters. Type III IFN, although signaling via a different surface receptor that is expressed mainly on epithelial cells, shares with type I IFN the same intracellular cascade [28]. Therefore, type I and type III IFNs protect host cells by inducing the expression of a plethora of ISG-encoding proteins interfering with viral replication and, thus, limiting both viral spread and viral load [29,30,31,32].

**Figure 2:**
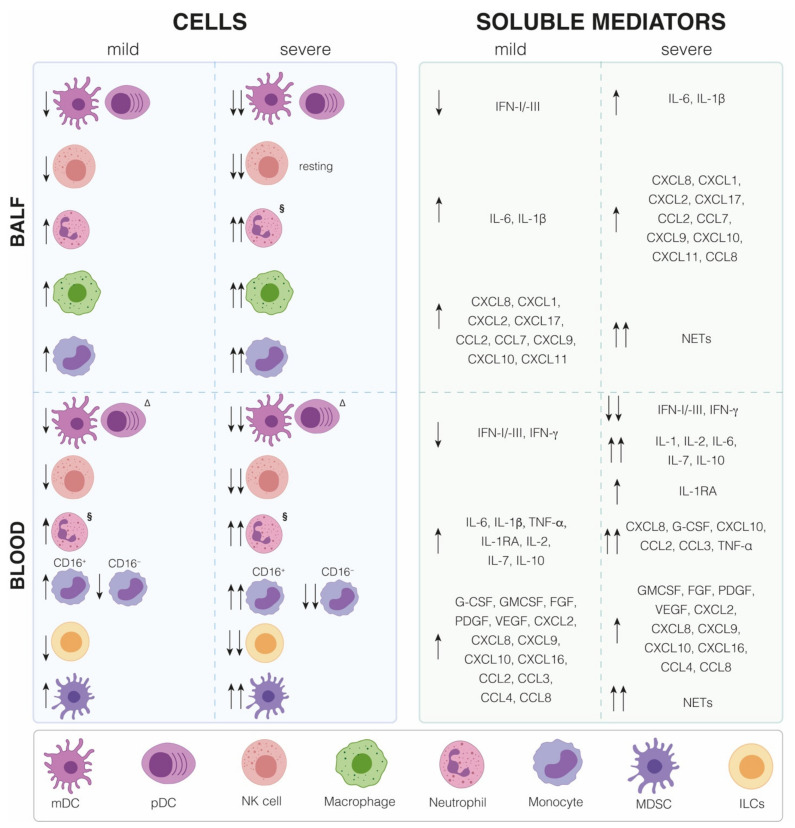
Immune cell distribution and cytokine/chemokine expression in COVID-19 patients. Distribution of innate immune cells and production of soluble mediators present in bronchoalveolar lavage fluid (BALFs) and blood samples from mild and severe COVID-19 patients with respect to healthy subjects. CCL: CC Chemokine ligand; CXCL: Chemokine (C-X-C motif) ligand; CD: Cluster of differentiation; IFN: Interferon; IL: Interleukin; FGF: Fibroblast growth factor; G-CSF: Granulocyte-colony stimulating factor; GMCSF: Granulocyte-macrophage colony-stimulating factor; NETs: Neutrophil extracellular traps; PDGF: Platelet-derived growth factor; VEGF: Vascular Endothelial growth factor.

The highly pathogenic potential of SARS-CoV-2 mainly relies on the peculiar ability to hamper the IFN pathway and, on the other hand, to stimulate an elevated production of proinflammatory chemokines and cytokines, in particular interleukin (IL)-6, thus suggesting that SARS-CoV-2 has developed multiple efficient mechanisms to shift the balance in favor of a proinflammatory status interfering with IFN production [33,34] (Figure 2). This also implies that, in a different manner from SARS-CoV, SARS-CoV-2 replicates more actively and effectively in human lung tissues, where a far higher viral load was found likely due to ongoing immune evasion mechanisms or defective viral clearance [34]. In addition, it has also been observed that the presence of neutralizing antibodies (Abs) targeting IFN-α2 and IFN-ω in about 10% of severe patients may contribute to an impaired antiviral gene expression [35]. In line with this evidence, no measurable IFN-β and low levels of IFN-α and ISGs were associated with a higher blood viral load and inflammatory response in sera of severe and critical COVID-19 patients as compared to mild cases [36] (Figure 2). The elevated ISG expression in peripheral blood mononuclear cells (PBMC) of mild or asymptomatic versus severe COVID-19 patients likely depends on an early and robust IFN production in the lungs that subsequently diffuses into the bloodstream where high IFN-α plasma levels are found [26,37,38]. Contoli et al. also reported that the reduced expression of type I IFN and high levels of proinflammatory cytokines in sera of severe COVID-19 patients reverse with the improvement of disease severity and inversely correlate with IL-10 level [37]. All these data indicate that the interplay between the virus replication and the IFN expression plays a critical role in the COVID-19 course and pose the basis for the therapeutic benefit of type I IFN administration in selected patients early in the course of SARS-CoV-2 infection, as a later time may turn detrimental [38,39].

### 4.2. Cytokine Storm

Viral infections can lead to immune system overactivation by inducing a massive release of proinflammatory cytokines. This phenomenon, known as cytokine storm, starts as a localized inflammatory response but then spreads systemically, further contributing to the recruitment of immune cells into infected tissues. A controlled cytokine release has a key role in resolving infection, but imbalanced levels of proinflammatory and antiviral mediators remain the main cause of ARDS and multiorgan failure [40,41,42]. The cytokine storm was not only observed in patients infected by SARS-CoV-2, which manifest severe symptoms, but also in SARS-CoV and MERS-CoV severe cases [43,44,45].

Several activated immune cells, namely B cells, T cells, NK cells, macrophages, DC, neutrophils, monocytes, and tissue-resident cells as epithelial and endothelial cells, contribute to the COVID-19 cytokine storm. In comparison to healthy controls, patients infected with SARS-CoV-2 show increased plasma levels of proinflammatory cytokines and chemokines, such as IL-1β, IL-1 receptor antagonist (IL1RA), IL-2, IL-6, IL-7, IL-10, tumor necrosis factor α (TNF-α), IFN-γ, granulocyte-macrophage colony-stimulating factor, granulocyte-colony stimulating factor (G-CSF), fibroblast growth factor, platelet-derived growth factor (PDGF), vascular endothelial growth factor, CC chemokine Ligand 2 (CCL2), CCL3, CCL4, chemokine (C-X-C motif) ligand 10 (CXCL10), CCL8, CXCL2, CXCL8, CXCL9, and CXCL16 (Figure 2). Moreover, a significant correlation between COVID-19 severity and the serum concentrations of IL-1, IL-2, IL-6, IL-7, IL-10, TNF-α, G-CSF, CCL2, CCL3, CXCL8, and CXCL10 has been observed [33,46,47,48,49]. Another complementary study has not only confirmed that severe COVID-19 patients display significantly higher plasma levels of IL-6, IL-10, and TNF-α than mild cases but also demonstrated a negative correlation between the plasma concentration of these cytokines and the recovery phase [50]. Interestingly, the elevated serum concentration of TNF-α, IL-6, and IL-10 detected in severe COVID-19 patients is associated with a reduced number of circulating T cells that, in addition, show an exhausted phenotype characterized by high expression of the immune checkpoint molecules programmed cell death protein 1 (PD-1) and T-cell immunoglobulin and mucin-domain containing-3 (Tim-3) [50]. An important feature of COVID-19 immunopathogenesis emerged from a longitudinal study conducted in patients with moderate and severe disease displaying a similar expression profiling of inflammatory cytokines up to 10 days after the disease onset, while, at later time points, TNF-α, IL-6, and IL-10 levels steadily declined in patients with moderate disease and instead remained elevated in those with severe COVID-19 [51]. Among the overexpressed cytokines and chemokines in COVID-19 patients, IL-6 represents a valuable biomarker due to the correlation of its plasma level observed in critical patients with both viral load and lung injury [52].

An interesting picture emerged from the analysis of the local cytokine/chemokine production present in bronchoalveolar lavage fluid (BALF) of SARS-CoV-2-infected individuals compared to healthy donors, confirming the inflammatory cytokine profile present in the blood (Figure 2). In particular, BALFs from severe or mild COVID-19 patients show the upregulation of IL-6, IL-1β, and several chemokines, such as CXCL8, CXCL1, and CXCL2, critical for neutrophils recruitment into the inflamed lung; CXCL17, CCL2, and CCL7, required for monocyte recruitment; and CXCL9, CXCL10, and CXCL11, which mediate T cell migration [53]. In addition, CCL2 and CCL8 were found elevated in postmortem lung specimens from COVID-19 patients compared to lung biopsies from healthy controls [33,54], thus corroborating the role of the cytokine storm in COVID-19 severity.

Thus, based on the current knowledge, the fine and concerted action of different soluble mediators, spanning from the antiviral and pro- and anti-inflammatory cytokines to different chemokines, contributes both to the fate of the infection as well as to local and systemic disease manifestations. 

### 4.3. Immune Evasion Strategies

**Figure 3:**
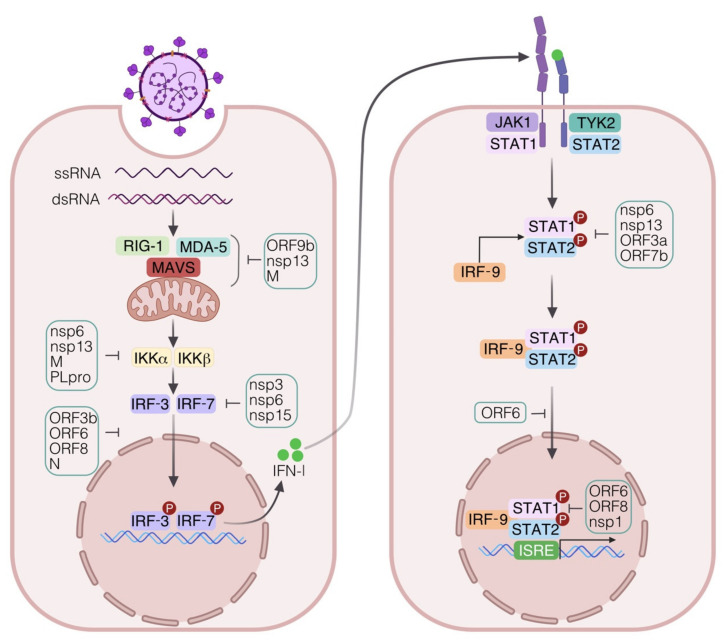
Strategies employed by SARS-CoV-2 to evade innate immune response. SARS-CoV-2 utilizes a plethora of structural and nonstructural proteins (nsp) to interfere with IFN signaling. In particular, the activation of the cytosolic RNA-sensing pathway is antagonized at multiple levels. SARS-CoV-2 nsp13, open reading frame (ORF)9b, and membrane (M) proteins target and associate with the cytosolic receptor MDA5 and with the adapter protein MAVS. The binding of nsp6 and nsp13 to the TANK binding kinase TBK1 causes the inhibition of IRF-3 phosphorylation and the consequent reduction of type I IFN release. TBK1 activity can also be affected by the ubiquitin-mediated degradation induced by SARS-CoV-2 M protein. Moreover, the papain-like protease (PLpro) impairs type I IFN response through the proteolytic cleavage of the ubiquitin-like molecule IFN-stimulated gene 15 (ISG15). The nuclear translocation of the IRF-3 transcription factor and the consequent activation of type I IFN pathway can be also prevented by ORF3b, ORF6, ORF8, and N proteins. SARS-CoV-2 nsp6, nsp13, ORF3a, and ORF7b proteins interfere with the phosphorylation/activation of both signal transducers and activators of transcription (STAT)1 and STAT2. The nuclear translocation of STAT1 may be inhibited by ORF6, while the ISRE promoting activity is hampered by ORF6, ORF8, and nsp1 proteins.

Coronaviruses have developed several strategies to escape the innate immune response by encoding a wide range of viral structural proteins and nsp that affects the IFN signaling pathway and, in turn, impairs the IFN-mediated antiviral responses [55] (Figure 3). In particular, with respect to the pathways regulating type I IFN expression, it has been demonstrated that the SARS-CoV-2 nsp6 and nsp13 inhibit IRF-3 phosphorylation by binding TANK binding kinase 1 (TBK1) and leading to a reduced IFN-β release, while the ORF6 prevents IRF-3 nuclear translocation by binding importin Karyopherin α2 [56,57]. During SARS-CoV-2 infection, the block of IRF-3 nuclear translocation and the consequent inefficient and delayed type I IFN response can be also due to the antagonistic function of the viral protein ORF3b or to the ubiquitin-mediated degradation of TBK1 promoted by M protein [58,59].

Moreover, ORF6, ORF8, and N proteins inhibit both IFN-β and NF-κB pathway activation, while ORF6, ORF8, and nsp1 impair the ISRE-driven transcription of ISGs [60]. The SARS-CoV-2-mediated suppression of type I IFN cascade is also carried out by ORF9b, nsp13, nsp1, and M proteins through the targeting of multiple components of the RIG-I/MDA-5/MAVS-signaling cascade including the direct association of these viral factors to the mitochondrial membrane with the adapter protein MAVS and with the translocase of the outer membrane 70 receptor [61,62,63,64].

Among SARS-CoV-2 factors, the papain-like protease, an essential viral enzyme involved in polyprotein processing, hampers IRF-3 phosphorylation, thus compromising type I IFN response through the proteolytic cleavage of the ubiquitin-like molecule ISG15 [65].

Regarding the viral proteins antagonizing type I IFN signaling, nsp6, nsp13, ORF3a, and ORF7b prevent both STAT1 and STAT2 phosphorylation [66]. Similarly, SARS-CoV-2 N, ORF6, and M proteins suppress the type I IFN pathway by blocking both STAT phosphorylation and their nuclear translocation [66,67].

Therefore, the multiple routes that SARS-CoV-2 exploits to elude the antiviral immunity clearly indicate not only the relevance of these responses for the early containment of the infection but also highlight the importance of acquiring a deeper knowledge of these immune evasion mechanisms to understand their contributions to COVID-19 pathogenesis and to develop efficient first-line drug therapeutics.

### 4.4. Inborn Genetic Defects

Considering that the evolution of COVID-19 pathogenesis profoundly depends on the efficiency of the host immune response, a few studies have also addressed the question of whether a “compromised” immune system might be a predisposing factor to SARS-CoV-2 infection [68,69,70]. A meta-analysis study reported that both immunosuppression and immunodeficiency are associated with an increased risk of severe COVID-19 disease [71]. Of note, a seminal paper by Zhang et al. demonstrated by using a genome-wide sequencing analysis that severe COVID-19 patients have inborn errors in TLR-3- and IRF7-dependent type I IFN immunity, with respect to those displaying asymptomatic infection, corroborating the crucial role of type I IFN in the control of SARS-CoV-2 infection [68]. In particular, at least 3.5% of patients with life-threatening COVID-19 pneumonia showed autosomal-recessive deficiencies in IRF-7 and IFNAR1 genes and autosomal-dominant deficiencies in those genes encoding TLR-3, Unc-93 homolog B1, TLR adapter molecule 1, TBK1, IRF-3, IRF-7, IFNAR1, and IFNAR2 [71]. These data together with results obtained by Bastard et al. showing the production of antitype I IFN autoantibodies in severe COVID-19 [35] confirm the importance of these antiviral cytokines in preventing the development of severe disease.

## 5. Cellular Mediators

**Figure 4:**
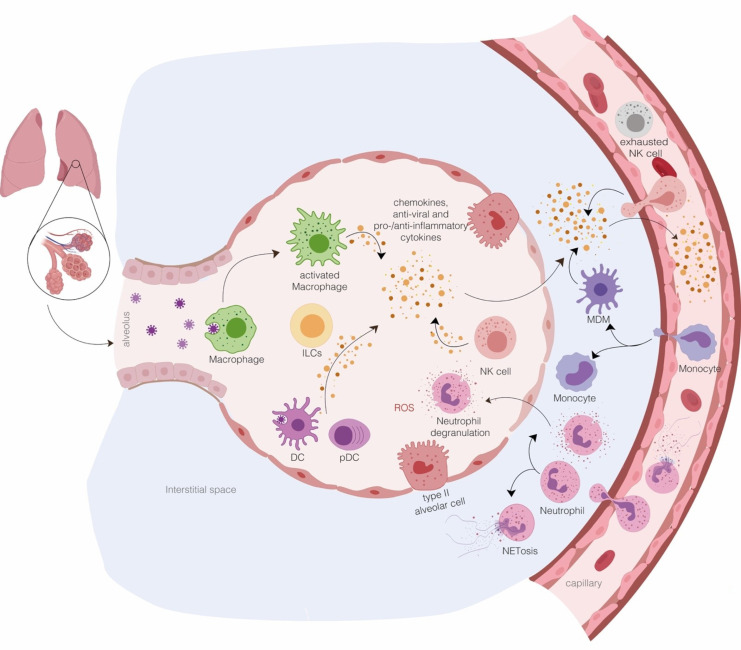
Innate immune response in SARS-CoV-2-infected lung. Aerosolized uptake of SARS-CoV-2 allows the infection of angiotensin-converting enzyme 2 (ACE2)-expressing target cells in the lung such as alveolar type II cells. A successful control of viral spread relies on the induction of local IFN-dependent antiviral state and the activity of alveolar macrophages that neutralize viruses and infected apoptotic cells and clear them by phagocytosis. The initial inflammation induced by the release of soluble factors, including proinflammatory cytokines and chemokines, from resident immune cells and infected epithelial cells attracts and activates neutrophils, monocytes, macrophages, dendritic cells (DC), natural killers (NK), and innate lymphoid cells into the site of infection, where they contribute to the elimination of the infected cells before virus spreading. If the virus takes over by dampening the IFN antiviral effect, the proinflammatory response increases because of the further infiltration of monocytes/macrophages, neutrophils, and several other adaptive immune cells from the bloodstream, resulting in “cytokine storms”. In addition, the formation of neutrophil extracellular traps (NETs)—aggregates composed by extracellular DNA fibers, histones, microbicidal proteins, and proteases released from the recruited neutrophils—can further trigger and sustain the local inflammation. Thus, the early phase of SARS-CoV-2 interaction with host cells, including innate immune cells, is crucial in determining the fate of the infection spanning from minimal lung damage with recovery and a maintaining of vascular integrity to the disruption of lung structure associated with pulmonary edema and pneumonia.

The initial viral recognition by tissue-resident immune cells triggers a local innate response that subsequently leads to the recruitment of other innate immune cells to carry out viral clearance (Figure 4).

A broad single-cell analysis was conducted by Liao et al. to characterize immune cells present in BALFs from COVID-19 patients with different degrees of disease severity in comparison to healthy individuals, and this study revealed interesting information on the local immune response to SARS-CoV-2 infection [72]. Macrophages, neutrophils, myeloid DC (mDC), pDC, NK cells, T cells, B cells, plasma cells, and epithelial cells were found in all the analyzed groups, although at different ratios according to disease severity. For instance, BALFs of SARS-CoV-2 affected patients showed a higher frequency of monocytes, macrophages, and neutrophils than healthy controls. When compared to mild cases, in BALFs from severe patients, a higher percentage of macrophages and neutrophils, but a smaller amount of mDC, pDC, T, and NK cells, was observed [72]. Therefore, it is conceivable that substantial recruitment to the lung of proinflammatory immune cells, particularly macrophages and neutrophils, might contribute especially in COVID-19 patients with severe symptoms to excessive inflammation resulting in systemic manifestations and multiorgan dysfunctions [72]. With this in mind, we focused our attention on cells participating in innate immune responses, namely monocytes, macrophages, DCs, NK, neutrophils, and ILCs and dissected their role in SARS-CoV-2-mediated disease (Figure 4).

### 5.1. Monocytes and Macrophages

It is known that myeloid cells are involved in the pathophysiology of coronavirus infection, either directly, as a virus target, or indirectly, as a producer of proinflammatory cytokines [73,74,75]. Indeed, the so-called macrophage activation syndrome characterized by SARS-CoV-2 hyperactivated macrophages has been associated with ARDS [76]. This evidence correlates with the finding that SARS-CoV-2 activates alveolar, splenic, and renal macrophages through ACE2 and enhances the secretion of IL-6, TNF-α, and IL-10 [77], even in the absence of a productive virus replication and generation of virus progenies [78,79].

The presence of monocytes and macrophages displaying a proinflammatory phenotype has been demonstrated in different localizations during COVID-19 [79,80]. Immunostaining of postmortem tissue from COVID-19 patients showed that in secondary lymph nodes, SARS-CoV-2 can infect ACE2-expressing resident CD169^+^ macrophages that, in turn, are stimulated to produce IL-6 levels [81].

In the peripheral blood of COVID-19 patients, where the total number of monocytes and macrophages was similar to healthy individuals [80], a high forward scatter monocyte population double-positive for CD14 and CD16 has been identified. Interestingly, this cell subset showed features of mixed M1/M2 macrophage polarization with higher expression of CD80 and CD206 and secretion of huge levels of IL-6, IL-10, and TNF-α as compared to normal controls [80]. Other studies, instead, reported that the number of CD14^high^CD16^-^ classical monocytes is decreased in COVID-19 patients, while the abundance of inflammatory CD14^+^CD16^+^, CD14^high^CD16^+^ intermediate, and CD14^+^CD16^high^ nonclassical monocytes increased according to the severity of COVID-19 [80,82,83]. In particular, patients with life-threatening COVID-19 display an enhanced absolute number of CD16^+^ monocytes with a significant decrease of CD16^-^ monocytes compared to both severe and mild patients [82]. Interestingly, Qin et al. showed that in CD16^+^ monocytes of critical COVID-19 patients, human leukocyte antigen (Ag)–DR isotype (HLA-DR) expression declines, while no significant changes in CD38 level were observed as compared to severe and mild patients [82]. However, in a counterintuitive manner, a positive correlation between IL-6 serum levels and CD16^+^ monocytes and an inverse correlation between HLA-DR molecules and IL-6 serum levels exist and were underlined in this study [82]. Moreover, few reports showed that monocytes from SARS-CoV-2-infected individuals possess a high surface expression of PD-1 ligand 1 (PD-L1), a suppressor marker, and low expression of the maturation marker CD80 [83,84]. Collectively, the emerging picture from the current knowledge shows that the alterations in monocyte phenotype observed in COVID-19 patients at different disease stages mainly result in reduced Ag presentation and dysfunctional immune response.

As anticipated previously, BALFs of SARS-CoV-2-affected patients show higher proportions of monocytes, macrophages, and neutrophils than healthy controls, expressing ficolin-1 (FCN1) and secreted phosphoprotein 1 (SPP1) markers and immunoregulatory genes [72], while fatty acid binding protein 4-expressing macrophages are preferentially contained in BALFs from patients with mild infection and healthy controls and completely lost in severely infected patients [72]. In addition, inflammatory monocytes, characterized by the expression of S100A8, S100A9, VCAN, FCN1, CD14, and CD62L, also display an IFN signature, which has been associated with a profibrosis differentiation pattern and were more abundant in severe than in mild COVID-19. This differential gene expression profile highlighted that monocyte-derived M1-like macrophages, producing highly inflammatory and potent chemokines, were abundant in BALFs from patients with severe COVID-19 as compared to mild cases or healthy controls. Conversely, a gene signature associated with reparative and profibrotic functions, characterizing the alternative M2-like macrophage phenotype, was present in mild COVID-19 cases [72].

Moreover, a very elegant picture of cytokine and chemokine production from monocyte-macrophages was obtained by single-cell analysis of BALFs from severe and mild COVID-19 [85]. In particular, in severe COVID-19 patients, monocyte-macrophages produced huge number of cytokines and chemokines but displayed a limited secretion of IFNs. Intriguingly, higher levels of anti-inflammatory molecules [IL-1 receptor type 2 (IL1R2), IL1RA, and transforming growth factor beta (TGF-β1)] and lower levels of IL-18 were also found in monocyte-macrophages from BALFs of severe COVID-19 compared with mild cases, whereas the classical proinflammatory cytokines (IL-1α, IL-1β, IL-6, and TNF-α) were detected at similar levels in the two groups. Regarding the production of monocyte- and neutrophil-recruiting chemokines, a high expression of CCL2, CCL3, CCL4, CCL7, CCL8, CXCL1, CXCL2, CXCL3, and CXCL8 was observed, while the T cell recruiting CXCL9 and CXCL16 chemokines were less expressed by monocyte-macrophages in BALFs of severe COVID-19 than those of mild cases [85]. Collectively, these data suggest that during severe COVID-19, lung monocyte-macrophages are prone to produce chemokines that recruit more monocytes and neutrophils, which, once migrated into the infected lung, contribute to the excessive production of proinflammatory cytokines [72] (Figure 4).

### 5.2. Dendritic Cells

DC are Ag-presenting cells (APC) that play a key role in activating both innate and adaptive immune responses. Three DC subpopulations are present in the lung: conventional DC (cDC), including the CD141^+^ cDC1 population (mainly activating a Th1 response); the CD1c^+^ cDC2 population (responsible for regulation and production of pro-inflammatory chemokines); and CD123^high^ pDC [86].

During viral infections, the functions of pDC are crucial as they are the main producer of the antiviral cytokine type I IFN [87]. Moving from this evidence, it was shown that BALFs from severe and critical COVID-19 patients contain a low number of pDC compared to subjects with moderate infection [72]. A similar picture was observed in blood samples from SARS-CoV-2-infected individuals, where the percentage of CD1c^+^ cDC, CD141^+^ cDC, and pDC populations was lower as compared to healthy controls [88]. In particular, in addition to the reduced pDC absolute count [30,36,85], Arunachalam et al. demonstrated that the functionality of pDC, in terms of IFN-α production, was impaired in SARS-CoV-2-infected patients compared to healthy individuals [89].

It has been also demonstrated that, in response to viral infection or single stimuli, pDC undergo phenotypical diversification [90]. They can diversify into three effector subpopulations: P1-pDCs (PD-L1^+^CD80^–^) specialized for type I IFN production, P2-pDCs (PD-L1^+^CD80^+^) exhibiting both innate and adaptive functions, and P3-pDCs (PD-L1^–^CD80^+^) that possesses adaptive functions [90]. By analyzing those pDC subpopulations, differences in pDC phenotype were also reported in hospitalized or asymptomatic subjects. In particular, pDC from asymptomatic COVID-19 patients mainly expressed PD-L1, while cells derived from severe COVID-19 patients were consistently represented by the PD-L1^+^CD80^+^ phenotype. The expression of costimulatory marker CD86 mirrored CD80 positivity in pDC of asymptomatic versus hospitalized patients, indicating that pDC are strongly activated to produce type I IFN during asymptomatic infection [26].

An in vitro study showed that isolated pDC, even if they do not express the ACE2 and TMPRSS2 receptors, and in absence of productive infection, are activated by SARS-CoV-2 and undergo diversification into P1, P2, and P3 subpopulations [91]. Moreover, SARS-CoV-2-stimulated pDC release high levels of type I and type III IFNs via TLR-7 pathway, while they do not produce TNF-α and IL-6 proinflammatory cytokines [26,91].

Moreover, SARS-CoV-2 patients also exhibit a significant decrease of peripheral mDC frequency compared to healthy controls [92]. Interestingly, the cDC:pDC ratio was significantly higher in the PBMC of severe patients than convalescent patients or healthy donors, thus representing a potential biomarker for severe COVID-19 [88]. Regarding DC functionality, it was shown that the expression level of the costimulatory and maturation-associated markers CD86 and CD80 was significantly lower, while that of the suppressive molecule PD-L1 was enhanced in COVID-19 patients with respect to healthy individuals [84,88]. These data have recently been confirmed by a paper by Saichi et al., who demonstrated by RNA-seq single cell analysis that circulating cDC1c^+^ DCs display an impaired capacity to mount the adaptive T cell-mediated response due to a reduced expression of the innate sensor CLEC9 and a decrease of major histocompatibility complex (MHC) class II-related genes and MHC class II transactivator activity [93].

Taken as a whole, this evidence indicates that SARS-CoV-2, by targeting the regulatory multitasking of the different APC subsets involved in the modulation of the inflammatory response, production of antiviral effector molecules, and priming an Ag-specific adaptive immune response, hits the main hub of the host immune response and breaks up the coordinated interplay between cellular and soluble mediators required to resolve the infection.

### 5.3. Natural Killer Cells

NK cells can control viral infections through the recognition and killing of virus infected cells by means of perforin-mediated cytotoxic activity or Ab-dependent cell-mediated cytotoxicity. However, in addition to killing activity, NK cells also possess immunomodulatory functions since they are able to suppress the inflammatory process triggered by viral infection in order to limit host damage and disease progression [94].

Human NK cells are divided into two subsets, namely the weakly cytolytic CD56^bright^CD16^-^ NK cells, predominant in tissues and specialized in cytokine production, and the CD56^dim^CD16^+^ NK cells that upon activation display potent cytolytic activity [94]. The latter, in particular, are characterized by the expression of the killer-immunoglobulin-like receptors (KIRs) which, together with CD16, are crucial for NK cytotoxic functions. In whole blood samples from SARS-CoV- and SARS-CoV-2-infected patients, the percentage of CD56^dim^CD16^+^KIR^+^ NK cells was significantly reduced, suggesting either impaired maturation or augmented recruitment of circulating NK cells into infected tissues [95,96]. In addition, it has been shown that SARS-CoV-2 infection influences NK-mediated cytotoxic activity by stimulating the expression of the inhibitory receptor NKG2A [97]. Indeed, in the NK of SARS-CoV-2-infected patients, the expression of the NKG2A receptor significantly increases compared to healthy controls, whereas the expression of the activation markers CD107a, IFN-γ, IL-2, and TNF-α diminishes. The unfavorable effect of SARS-CoV-2 on NK activation status and cytolytic activity also correlates to the increased expression of genes encoding the inhibitory receptors lymphocyte activating-3 and Tim-3 [36,98]. Interestingly, alterations in NK number and NKG2A expression seen in COVID-19 patients were restored in convalescent patients [97,99,100].

The reduced peripheral NK cell counts and impaired cytotoxic activity observed in severe SARS-CoV-2-infected subjects, with respect to mild cases [47,85,88,99,100,101] and in deceased versus survivor patients [102], significantly parallel the increase in IL-6 circulating levels, suggesting that the functional impairment of NK activity leads to enhanced innate immune cell activation with massive proinflammatory cytokine production [67,103]. Thus, the phenotype acquired by NK cells at the early stage of SARS-CoV-2 infection delineates the functional exhaustion of cytotoxic and immunoregulatory activity of NK and correlates to disease progression.

Moving into the alveolar compartment, while a significant reduction of resting NK cells in the absence of significant changes in activated NK cells has been observed in the BALFs of COVID-19 subjects [53], another study showed that the percentage of NK cells in the BALFs of patients with severe COVID-19 was higher than those present in subjects with moderate infection or in healthy donors [72]. These data on the presence of NK cells in the infected tissues are still puzzling and often discordant, probably due to differences in the timing of sample collection or to disease severity. Thus, further analysis is required.

### 5.4. Neutrophils

Neutrophils are recruited to infection sites by different signals, including pathogen engulfment, reactive oxygen species formation, and degranulation but also through the formation of neutrophil extracellular traps (NETs). Compared to mild SARS-CoV-2-infected individuals, blood samples from severe patients are characterized by a high neutrophil-to-lymphocyte ratio (NLR), a widely used marker of inflammation and infection [47,104,105]. Accordingly, it has been observed that in deceased patients, neutrophil count increased while lymphocyte number lowered compared to survivors [102,106]. Neutrophils of COVID-19 patients also seem to possess reduced Ag-presentation capacity, due to the impaired HLA-DR and increased PD-L1 surface expression as compared to healthy donors [84]. Moreover, in COVID-19 patients, neutrophils act as a hyperinflammation driver by means of enhanced cytokine production and cell degranulation [84]. Accordingly, the strong local inflammatory response leading to tissue injury is dependent from the neutrophil recruitment mediated by some chemokines upregulated in both BALF and blood samples, namely CXCL1, CXCL2, CXCL8, CXCL10, CCL2, and CCL7 [53,54,106]. In line with this observation, the BALFs of COVID-19 patients are also enriched in neutrophils—high numbers in severe cases—and possess a high NLR with respect to healthy controls [53,72]. Interestingly, Combes et al. showed that a neutrophil subpopulation expressing an ISG signature was strongly represented in patients with mild/moderate COVID-19 but not in patients with severe COVID-19 [105].

Once recruited into the lung, neutrophils may participate in COVID-19 pathological manifestations through the formation of NETs, aggregates composed by extracellular DNA fibers, histones, microbicidal proteins, and proteases, whose function is the binding and killing of extracellular pathogens including viruses [107,108]. However, excessive NET formation can trigger inflammation and thrombosis, which results in permanent organ damage [107,108,109]. NET dysregulation was found in COVID-19 patients who showed elevated serum levels of cell-free DNA, myeloperoxidase DNA, and citrullinated histone H3 [110]. Accordingly, the transcriptional profile from BALF and lung specimens showed an induction of NET-associated genes in SARS-CoV-2-infected patients with respect to healthy controls [111].

Interestingly, in vitro experiments demonstrated that the exposition of neutrophils from healthy controls to COVID-19 patient sera promotes NET release [112]. This evidence corroborates the hypothesis on the pathological role of excessive NET formation in severe COVID-19 cases and points to NETs as a potential target for severe COVID-19.

Neutrophils share their origin and morphological/phenotypical features with polymorphonuclear myeloid-derived suppressor cells (PMN-MDSC). Nonetheless, in contrast to the protective role of neutrophils against invading pathogens, PMN-MDSC play a detrimental and immunosuppressive activity in several infectious diseases since they are able to suppress T-cell functions, dampening excessive immune response via TGF-β production [113]. Interestingly, the significant expansion of PMN-MDSC has recently been reported in COVID-19 patients requiring intensive care treatment, and the association between PMN-MDCS abundance and COVID-19 progression has been proposed as a biomarker of disease severity [114,115].

### 5.5. Innate Lymphoid Cells

ILCs are a relatively new and growing family of tissue-resident (mainly lung and mucosal tissue) immune cells belonging to lymphoid lineage, but, different from T cells, they do not express Ag-specific receptors and do not undergo clonal selection and expansion following stimulation [116,117]. Similar to conventional innate cells, ILCs rapidly respond to tissue injury and microbial insults by secreting T cell-like inflammatory cytokines able to regulate homeostasis and inflammation and to direct the subsequent immune response against the triggering and harmful stimulus [116,117]. In particular, these non-T cell receptor immune cells can be classified on the basis of their cytokinome in group 1 ILC (ILC1), paralleling Th1 secretome; group 2 ILC (ILC2), releasing the Th2 cytokine IL-5 and IL-13; and group 3 ILC (ILC3), more highly linked to Th17 lineage and able to produce both IL-17 and IL-22 [118]. The importance of the ILC3 subset in the context of viral infection has recently been reported by demonstrating that the presence of IL-22 can limit tissue damage and prevent secondary bacterial infections in the Influenza (Flu) disease [119,120,121]. Given the similarity between COVID-19 and the Flu for the risk of the exacerbation of secondary bacterial infection, a beneficial effect of IL-22 in limiting disease severity has also recently been proposed for SARS-CoV-2 infection [118]. Moreover, it has been reported that the number of ILC1, ILC2, and ILC precursor cells decreases with the concurrent disease severity [122]. In ILCs of severe COVID-19 patients, the expression of the tissue homing receptor CD69 was found to be elevated, indicating that homing to the lung is favored in severe cases and suggesting ILC blood level as a prognostic marker of SARS-CoV-2 infection [123].

## 6. Conclusions

The knowledge accumulated so far concerning the ongoing pandemic indicates that COVID-19 severity and the associated mortality rate derive either from a dysregulated immunopathology induced directly by the infection or by the tissue damage caused by the immune response to viral replication. Based on this finding, the altered immune response represents the first target for therapeutic interventions aimed at modifying the immunopathogenesis of SARS-CoV-2 infection. Nevertheless, differences in the immunopathogenic mechanisms of COVID-19 manifestation among infected subjects contribute to complicating our understanding of the disease and the development of a common win-win strategy.

Moreover, given the rapidly evolving scenario due to ongoing vaccination campaigns and the emergence of SARS-CoV-2 variants, future studies are still required to achieve a more complete view of the multifaceted interaction of the human host with this virus that will live with us for at least the near future.

## Data Availability

Not applicable.

## Abbreviations

Ag: antigen; APC: antigen presenting cells; ACE2: angiotensin-converting enzyme 2; ARDS: acute respiratory distress syndrome; BALF: bronchoalveolar lavage fluid; CCL: C-C motif chemokine ligand; cDC: conventional dendritic cells; COVID-19: coronavirus disease 2019; CXCL: C-X-C motif chemokine ligand; DC: dendritic cells; dsRNA: double-stranded RNA; E: envelope protein; ERGIC: endoplasmic reticulum-Golgi compartment; FCN1: ficolin 1; Flu: influenza; G-CSF: granulocyte colony-stimulating factor; HLA-DR: human leukocyte antigen–DR isotype; IFN: interferon; IFNAR: interferon alpha/beta receptor; IL: interleukin; IL1R: IL-1 receptor; IL1RA: IL-1 receptor antagonist; ILCs: innate lymphoid cells; IKKα: I-Kappa-B Kinase alpha; IKKβ: I-Kappa-B Kinase beta; IKKε: I-Kappa-B kinase epsilon; IRF: interferon regulatory factor; ISGs: interferon stimulated genes; ISRE: IFN-stimulated response elements; KIR: killer cell immunoglobulin-like receptors; M: membrane protein; MAVS: adapter protein mitochondrial antiviral signaling protein; MDA-5: melanoma differentiation-associated protein 5; mDC: myeloid dendritic cells; MERS-CoV: Middle East respiratory syndrome coronavirus; MHC: major histocompatibility complex; MyD88: myeloid differentiation factor 88; N: nucleocapsid protein; NET: neutrophil extracellular traps; NF-κB: nuclear factor kappa light-chain-enhancer of activated B cells; NK: natural killer cells; NLR: neutrophil-to-lymphocyte ratio; nsp: nonstructural proteins; ORF: open reading frame; PBMC: peripheral blood mononuclear cell; PD-1: programmed death-1; pDCs: plasmacytoid dendritic cells; PDL-1: programmed death-ligand 1; PMN-MDSC: polymorphonuclear myeloid-derived suppressor cells; PRRs: pattern recognition receptors; RIG-I: retinoic acid-inducible gene I; RNA: ribonucleic acid; S: spike glycoprotein; SARS-CoV: severe acute respiratory syndrome coronavirus; SPP1: secreted phosphoprotein 1; ssRNA: single-stranded RNA; STAT: signal transducer and activator of transcription; TBK1: TANK binding kinase 1; TGF-β1: transforming growth factor beta 1; Th: T helper cells; Tim-3: T cell immunoglobulin and mucin domain-containing protein 3; TIR: Toll-interleukin receptor; TIRAP: TIR domain-containing adapter protein; TLR: Toll-like receptors; TMPRSS2: transmembrane serine protease 2; TNF: tumor necrosis factor; TRIF: TIR domain-containing adapter protein inducing interferon (IFN)-β.
